# Complete mitochondrial genome and phylogenetic analysis of brown alga *Scytosiphon lomentaria* (Ectocarpales, Phaeophyceae)

**DOI:** 10.1080/23802359.2018.1536448

**Published:** 2018-11-25

**Authors:** Yu Zang, Jun Chen, You Xia, Bin Zhou

**Affiliations:** College of Marine Life Sciences, Ocean University of China, Qingdao, China

**Keywords:** Mitochondrial genome, phylogenetic analysis, *Scytosiphon lomentaria*

## Abstract

The present study provides a mitochondrial genome sequence of *Scytosiphon lomentaria*, which is better than the previously established data in China. This mitogenome is 39,971 bp in length and consists of 35 protein-coding genes, 3 ribosomal-RNA genes, 24 transfer-RNA genes, and 5 open reading frames. Overall, the base composition was as follows: A, 29.7%; C, 12.8%; G, 19.2%; and T, 38.4%. The phylogenetic analysis based on the protein-coding genes indicated that the *S. lomentaria* collected in the samples was at an earlier differentiation position. These results contribute to the further understanding of Phaeophyceae phylogenetic relationships and species identification.

*Scytosiphon lomentaria* is a common brown seaweed, widely distributed in cold and warm waters around the world (Mann 1991; Kogame et al. 2005). This species belongs to the family Phaeophyceae of the order Ectocarpales, and grows attached to shells and stones in intertidal or shallow subtidal zones. Previous studies on its morphogenesis, life history, and molecular phylogenesis have suggested that the population of this species in different geographical areas vary substantially (Kogame, Rindi, et al. 2015). Earlier phylogenetic analyses based on the mitochondrial *cox3* and the nuclear ribosomal ITS divided the North-East Atlantic and the Mediterranean populations into four separate well-supported clades (Kogame, Ishikawa, et al. 2015). Due to its delicious taste and rich nutritive value, *S. lomentaria* is a favorite food for the coastal residents of North-East Asia (Sahoo and Seckbach 2015). However, the quantity of *S. lomentaria* has been considerably reduced due to ecological damage and changes in the environmental factors. Establishing a complete mitochondrial genome sequence will contribute to the development of a strategy for the conservation of this important macroalgae.

In the present study, the complete mitochondrial genome of *S. lomentaria* was initially assembled from previously published Illumina sequencing data (SRR5026350), used as a PCR primer guide. These specimens were collected from the beach of Xinghai Bay, China. The whole mitochondrial genome was revised with 48 pairs of primers, which were amplified and sequenced by protocols in Gene Denovo Laboratory, where the specimen and DNA were stored. The length of the complete mitochondrial genome of *S. lomentaria* is 39,971 bp, with an A + T content of 68.1% and the following base composition: A (29.7%), C (12.8%), G (19.2%), and T (38.4%). The genome comprised 67 genes, including 35 protein-coding genes (PCGs), 24 tRNAs, 3 rRNAs, and 5 open reading frames (ORFs). The 24 tRNA genes were as follows: *trnK*(*uuu*), *trnA*(*ugc*), *trnD*(*guc*), *trnM*(*cau*), *trnL*(*uaa*), *trnH*(*gug*), *trnC*(*gca*), *trnN*(*guu*), *trnF*(*gaa*), *trnW*(*cca*), *trnM*(*cau*), *trnQ*(*uug*), *trnL*(*uag*), *trnL*(*caa*), *trnG*(*gcc*), *trnY*(*gua*), *trnR*(*ucu*), *trnI*(*gau*), *trnE*(*uuc*), *trnQ*(*cug*), *trnS*(*gcu*), *tRNA-Ser*, *trnM*(*cau)*, *trnP*(*ugg*). The 35 protein-coding genes include (*rps2*-*4*, *7*, *8*, *10*-*14*, and *19*; *rpl2*, *5*, *6*, *14*, *16*, *31*, and *1*-*7*, *4L*, *9*, *11*, *cob*, *cox1*-*3*, *atp6*, *8*, *9*, and *tatC*). Five ORFs contained ORF78, ORF40, ORF127, ORF227, and ORF757. Compared with the only other *S. lomentaria* mitogenome established in Sanggou Bay, China (Liu and Pang 2014), despite the identical number of annotation genes, there was an increase in the length of 3,053 bp, 1 ORF, and 1 tRNA less in the new *S. lomentaria* mitogenome sequenced by us.

The phylogenetic relationship of *S. lomentaria* and other brown algae species was inferred using the neighbor-joining method in MEGA 7.0 (Kumar et al. 2016). The results showed that the species of Phaeophyceae were gathered in the same branch. *Scytosiphon lomentaria* was at an earlier differentiation position, and the distance between *S. lomentaria* and *Undaria pinnatifida* was nearer compared to those between other species of Phaeophyceae (Figure 1). These new mitochondrial genome data are more complete and can be better used to provide a basis for studies of the mitochondrial evolution of Phaeophyceae.

**Figure 1.**
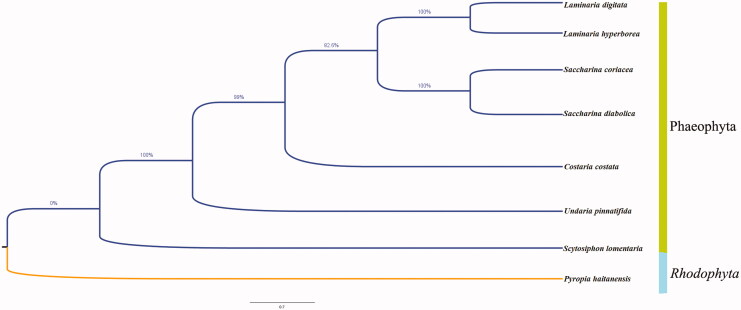
Neighbor-joining phylogenetic tree of the *Scytosiphon lomentaria* and seven other species based on the concatenated nucleotide sequences of 13 protein-coding genes. Numbers on nodes indicate bootstrap support value, based on 500 replicates.
